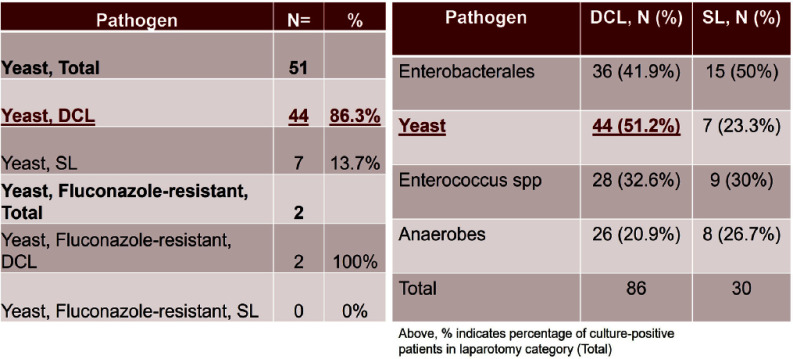# Incidence and Microbiology of Infectious Complications in Civilian Trauma Laparotomy Patients

**DOI:** 10.1017/ash.2025.253

**Published:** 2025-09-24

**Authors:** Eric Roessler, Andrew Benjamin, Divya Harsha Manukonda, Jennifer Pisano, Elizabeth Bell

**Affiliations:** 1 University of Chicago; 2 University of Chicago; 3 Center for Research Informatics; 4 University of Chicago Medicine; 5 University of Chicago

## Abstract

**Background:** Infection is a common and highly morbid postoperative complication in victims of physical trauma. Current literature analyzing the infectious sequelae of physical trauma predominately comes from military data, where blast trauma, rather than blunt or penetrating trauma, is most common. The epidemiology and management of infectious sequelae of civilian trauma are poorly understood, as is perioperative antimicrobial management of trauma laparotomy. **Methods:** We performed a single-center retrospective chart review using data from University of Chicago’s electronic medical record (Epic) and the National Trauma Registry. Patients 16 years and older admitted for level 1-2 trauma who underwent laparotomy between 5/1/2018-3/18/2023 were included. Using informatics and manual chart review, we analyzed patient demographics, rates of infection, sites of infection, timing of infection from initial trauma event, and causative organisms. We compared patients based on mechanism of injury (blunt versus penetrating) and whether patients underwent damage control laparotomy (DCL)--where the abdomen is left in discontinuity after the initial laparotomy--or single laparotomy (SL). **Results:** 430 patients met criteria. The median age was 30. Patients were majority Black (80.9%) and male (80.9%). 80.5% of patients had penetrating trauma, of which 90% were gunshot wounds (GSW). 19.8% had blunt trauma, of which 89% were motor-vehicle crashes (MVC). 19 (4.4%) died during initial stabilization, 199 (46.3%) underwent single laparotomy, and 212 (49.3%) underwent DCL (Figure 1). Of patients that survived initial stabilization, 27 (6.6%) developed a bloodstream infection (BSI), of which 21 (77.8%) came from the DCL group (Figures 2, 3). 19% of BSI in the DCL group were caused by yeast. 30.7% of patients developed a culture-positive surgical site infection (SSI) or intra-abdominal infection (IAI), with a rate of 40.6% in the DCL group (Table 2). Yeast were isolated in 40.5% of patients with positive cultures, 86.3% of which were isolated in the DCL group, with an overall incidence of 20.8% in the entire DCL group. Median time from arrival to infection diagnosis was 11 days. Patients generally received empiric Piperacillin-tazobactam while the abdomen was in discontinuity. **Conclusions:** Infection in civilian trauma laparotomy often arises as SSI or IAI, and is most pronounced in the DCL population. Yeast represents an unexpectedly high proportion of causative organisms. Further research is required to assess whether yeast burden can be mitigated by either incorporating antifungal prophylaxis at time of initial laparotomy, or by shortening empiric post-laparotomy antibiotic courses.

**Figure:**
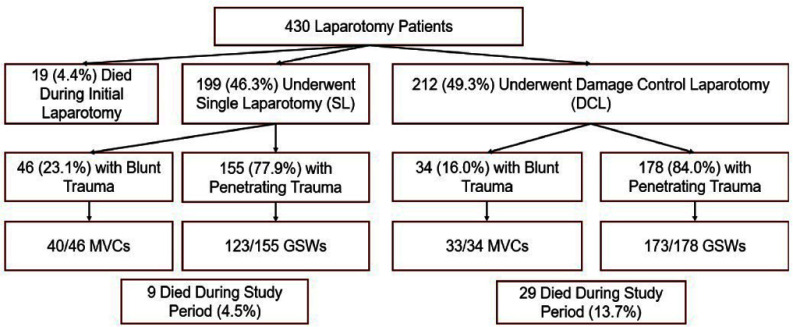

**Figure:**
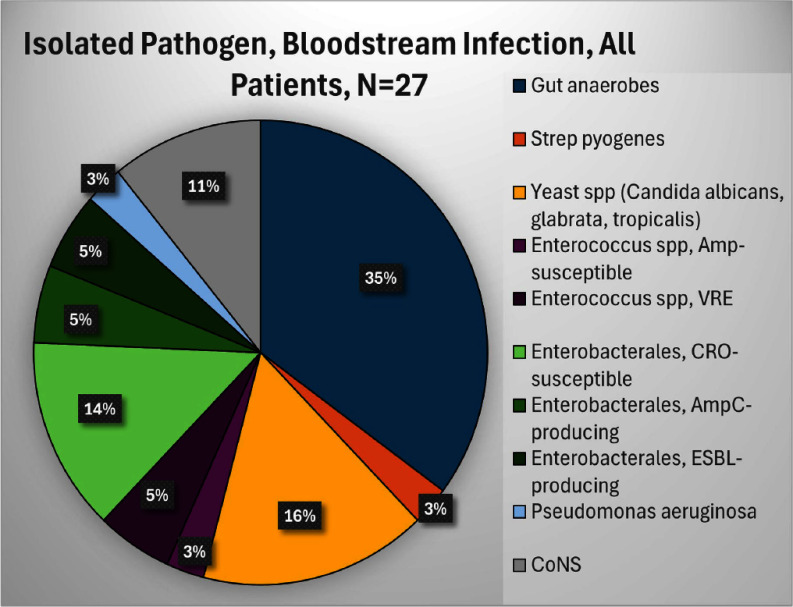

**Figure:**
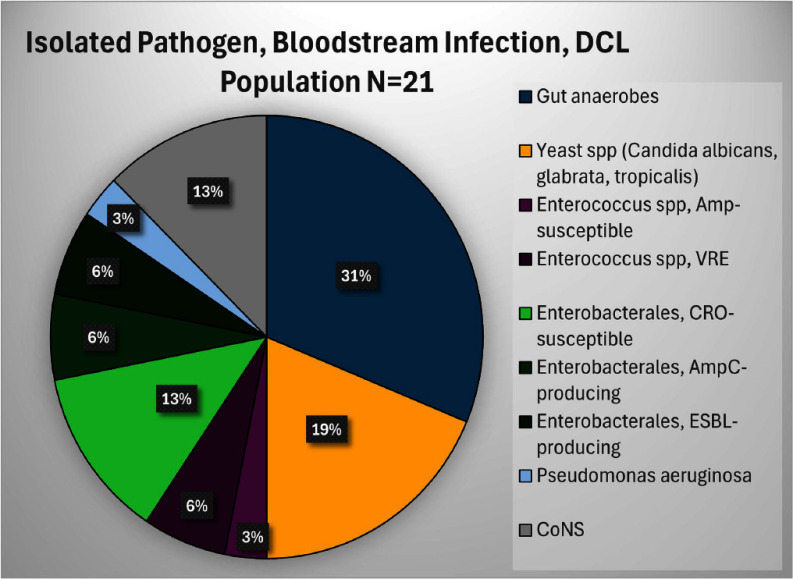

**Figure:**
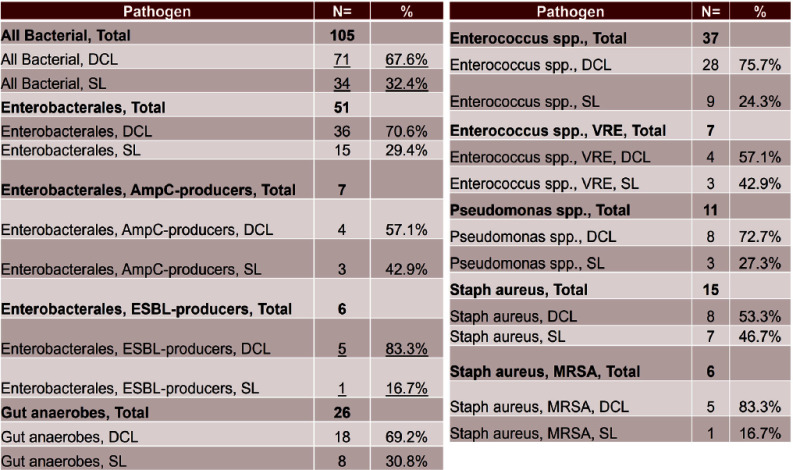

**Figure:**